# Student perspectives on interdisciplinary learning in public health education: insights from a mixed-methods study

**DOI:** 10.3389/fpubh.2024.1516525

**Published:** 2024-12-10

**Authors:** Raymond Boon Tar Lim, Claire Gek Ling Tan, Kelly Voo, Yock Leng Lee, Cecilia Woon Chien Teng

**Affiliations:** Saw Swee Hock School of Public Health, National University of Singapore and National University Health System, Singapore, Singapore

**Keywords:** evaluation, interdisciplinary learning, public health, student perceptions, mixed methods study, higher education

## Abstract

Interdisciplinary learning is essential for equipping future health professionals to tackle the complexities of contemporary public health. This mixed-methods study investigates the experiences and perspectives of undergraduate public health students in Singapore on interdisciplinary learning in higher education and employed a convergent parallel design by combining a cross-sectional survey with in-depth interviews. Quantitative data were used to assess the relationship between students’ exposure to interdisciplinary learning and its perceived value, while qualitative analysis explored key themes related to facilitators and barriers. Among 52 survey respondents and 11 interview participants, nearly half regularly engaged in interdisciplinary learning. Students with greater exposure to interdisciplinary experiences demonstrated more positive perceptions. Facilitators identified included career development opportunities, faculty engagement, and diverse learning experiences. Barriers such as disciplinary disconnects and the challenge of managing varied skill sets were also highlighted. This study provides valuable insights into interdisciplinary learning in higher education pertaining to public health, particularly within the context of health professions training. The findings suggest that enhancing curricular design, integrating interdisciplinary approaches more effectively, and increasing faculty support can address identified barriers and better prepare students for the demands of their future public health careers.

## Introduction

Interdisciplinary education is defined as the integration of knowledge from multiple disciplines to achieve outcomes that cannot be realised through a singular disciplinary approach (1). This approach involves blending and linking various epistemological forms and synthesising insights from multiple fields, resulting in curricula that draw upon two or more areas of study. In higher education for health professions, this approach plays a critical role in preparing future professionals to address multifaceted public health challenges. By blending various epistemological forms and synthesising insights from multiple fields, interdisciplinary education equips students with the skills needed to navigate the complexities of modern healthcare. As health professions increasingly require collaboration across disciplines, curricula that incorporate multiple areas of study, such as public health, environmental health, and social sciences, become essential (2).

The importance of interdisciplinary education in health professions is evident, as many core subjects—such as research methods, health policy, and organisational management—are inherently interdisciplinary (3). Moreover, employers in the health sector seek graduates who possess not only technical expertise but also critical soft skills, such as complex problem-solving, critical thinking, and cognitive flexibility (4). The growing emphasis on emotional intelligence and the ability to work across diverse disciplines highlights the importance of interdisciplinary education in preparing health professionals for modern workforce demands (4).

Previous research has highlighted the need for better integration of interdisciplinary knowledge and competencies within higher education pertaining to health professions (5). Several challenges in implementing interdisciplinary education have been identified, including lack of intrinsic motivation, inadequate learning environments, and reliance on traditional teaching methods that may not effectively support interdisciplinary learning (6). Despite this, significant gaps in the literature remain unaddressed.

Firstly, existing research predominantly focuses on clinical disciplines such as medicine, nursing, and allied health (7–9). These studies have provided valuable insights into how interdisciplinary approaches can enhance clinical practice and patient care. However, the findings from these clinical settings may not be directly transferable to public health contexts due to the distinct nature of public health education and practice. In clinical disciplines, interdisciplinary learning often revolves around collaborative patient care and the integration of diverse medical specialisations to address immediate health needs. In contrast, public health education focuses on broader, population-level issues such as disease prevention, health promotion, and policy development. Interdisciplinary approaches in public health must therefore address distinct challenges, such as integrating knowledge from diverse fields, including economics, social sciences, environmental health, and policy studies—areas that are less prominent in clinical settings (10). Therefore, there is a critical need for research that explores how interdisciplinary learning is implemented in public health programmes within the broader context of higher education pertaining to health professions.

Secondly, much of the current literature on interdisciplinary education in public health focuses on postgraduate levels. However, there has been a notable increase in undergraduate public health programmes globally, with a significant rise in public health degree conferrals in recent years (11). This growth necessitates research into how undergraduate public health programmes incorporate interdisciplinary learning and how such integration influences students’ educational experiences and outcomes. This gap is particularly pertinent given that students in undergraduate public health programmes often come from diverse backgrounds (12).

Thirdly, the literature often prioritises curriculum design and institutional outcomes over students’ perspectives (13). Understanding students’ perceptions and experiences with interdisciplinary learning is crucial for improving educational practices in health professions. Although facilitators of interdisciplinary learning have been identified in various contexts (14), empirical data on undergraduate public health students’ experiences remain limited. As future health professionals, these students’ insights are invaluable in refining educational practices to meet the evolving demands of the healthcare industry (15).

Singapore boasts a well-developed healthcare system and places an increasing emphasis on higher education pertaining to health professions. The National University of Singapore (NUS) offers undergraduate public health programmes through the country’s only national school of public health, the Saw Swee Hock School of Public Health (SSHSPH). These programmes include a minor (introduced in 2013) and a second major (introduced in 2021) in public health. This study aims to address gaps in the literature by exploring student perspectives on interdisciplinary learning in undergraduate public health programmes. Specifically, it seeks to (i) assess students’ perceptions of interdisciplinary learning within these programmes, (ii) describe their experiences, and (iii) explore the facilitators and barriers to effective interdisciplinary education.

## Materials and methods

### Study design and setting

We used a mixed methods approach that combined a cross-sectional survey (quantitative phase) with in-depth interviews (IDIs) (qualitative phase). This approach utilised a convergent parallel design, which involves collecting and analysing qualitative and quantitative data separately before comparing and relating the results. The goal was to create complementary datasets that inform and enrich each other. We examined areas of convergence and divergence between the qualitative and quantitative findings to provide a comprehensive understanding of the research topic. The entire study was conducted at SSHSPH, NUS.

### Quantitative phase

The cross-sectional survey was conducted from January 2023 to September 2023. Recruitment was carried out through an open online invitation, including a mass email sent to all students enrolled in the Minor and Second Major in Public Health programmes at SSHSPH, NUS. Participants were required to meet the inclusion criterion of being an undergraduate student currently enrolled in either the Public Health Minor or Public Health Second Major programme at NUS.

### Survey questionnaire

The questionnaire was designed to be anonymous and administered online. It included questions on sociodemographic characteristics, the extent of exposure to interdisciplinary learning, and modified items assessing students’ perceptions of interdisciplinary learning, using the Interdisciplinary Education Perception Scale (16). The questionnaire provided a definition of interdisciplinary learning, describing it as a method of learning that integrates knowledge and modes of thinking from two or more disciplines or established areas of expertise to achieve a cognitive advancement—such as explaining a phenomenon, solving a problem, or creating a product—in ways that would have been impossible or unlikely through single-disciplinary means (17).

To assess students’ exposure to interdisciplinary learning, respondents answered the following question: *“Consider all the learning experiences you have had so far during your first major and Public Health Minor/Second Major programmes. To what extent have these experiences involved interdisciplinary learning between the public health curriculum and your first major?”* This question used a six-point Likert scale ranging from *“No exposure (e.g., No occasion in a semester)”* to *“Very high exposure (e.g., More than 7 occasions in a semester).”* Responses of *“No,” “Minimal,” and “Some”* were classified as infrequent, while *“Moderate,” “High,” and “Very High”* were classified as frequent.

Perceptions of interdisciplinary learning were measured using a five-point Likert scale (Not at all, To a low extent, To some extent, To a high extent, To a very high extent). These responses were later dichotomised into *“Disagree/Neutral”* (Not at all, To a low extent, To some extent) and *“Agree”* (To a high extent, To a very high extent) for meaningful analysis.

To minimise social desirability biases, several measures were implemented: (i) the questionnaire was self-administered online, (ii) we emphasised the importance of providing honest responses for programme improvement, and (iii) the questionnaire was worded in a non-judgemental manner (e.g., use of neutral wording).

### Statistical analysis

The prevalence of participants with frequent versus infrequent exposure to interdisciplinary learning was assessed. Bivariate analyses were conducted to explore the relationship between the frequency of exposure and students’ perceptions of interdisciplinary learning. Chi-square tests were performed to assess whether there were significant differences in perceptions between the two exposure groups. If any expected frequency in the contingency table was below 5, Fisher’s exact test was applied as an alternative to ensure the validity of the results. The significance level for all tests was set at *p* < 0.05. Descriptive statistics (such as frequencies and percentages) were used to summarise participant characteristics and exposure groups. All statistical analyses were conducted using STATA version 15.0 (Stata Corp, College Station, TX, USA).

### Qualitative phase

In the qualitative phase, students were purposively selected for IDIs using maximum variation sampling. The selection criteria were based on participants who had taken part in the quantitative phase of the study, expressed interest in the qualitative phase, and provided their contact details. From this group, participants were selected to represent a variety of characteristics, including differences in exposure to interdisciplinary learning, sex, faculty/school, and year of study, to capture a wide range of experiences and viewpoints. The interviews, each lasting approximately 1 h, were conducted either face-to-face or online between February 2023 and October 2023. A topic guide was used during the interviews to gain insights into students’ experiences and to identify both facilitators and barriers to interdisciplinary learning.

### Qualitative data analysis

The interviews were transcribed verbatim and checked for accuracy against the recordings. These were then imported into NVivo 11.0 and coded line-by-line. The third and fourth authors coded and analysed the data in parallel, independently. The qualitative data were analysed using inductive thematic analysis, following the six-step procedure outlined by Braun and Clarke (18), to identify key themes and patterns. The transcripts were read multiple times to enhance familiarity with the content.

Initial codes were generated independently by the third and fourth author. Both authors then met to establish inter-coder reliability by comparing their coded transcripts and discussing any discrepancies. This iterative process involved calculating a Cohen’s Kappa score for each round of coding to quantitatively assess agreement. Discrepancies were resolved through discussion, leading to iterative refinements of the codebook. New codes were added as needed based on the emerging data, and coding guidelines were clarified to improve consistency. The inter-coder reliability threshold was set at a Cohen’s Kappa of 0.80, indicating substantial agreement, which was achieved by the sixth transcript. Once reliability was established, the finalised codebook was applied to code the remaining transcripts.

Following coding, the codes were independently categorised and condensed into preliminary themes and subthemes by the same two authors. Discrepancies in theme categorisation were then resolved through consensus, with input from the first two authors.

## Results

### Quantitative results

A total of 52 students participated in the survey. Table 1 presents the demographic characteristics of these participants, categorised by their frequency of exposure to interdisciplinary learning. The prevalence of frequent exposure to interdisciplinary learning among participants was 46.2%.

**Table 1:** Demographic characteristics of survey participants based on frequency of exposure to interdisciplinary learning.

Demographic characteristic	Total	Infrequent exposure	Frequent exposure
	(*N* = 52)	(*N* = 28)	(*N* = 24)
	*n* (%)	*n* (%)	*n* (%)
Sex			
Male	19 (36.5)	13 (46.4)	6 (25.0)
Female	33 (63.5)	15 (53.6)	18 (75.0)
Year of study			
Junior (Year 1 and 2)	27 (51.9)	14 (50.0)	13 (54.2)
Senior (Year 3 and above)	25 (48.1)	14 (50.0)	11 (45.8)
Faculty/school			
Non-science	26 (50.0)	20 (71.4)	6 (25.0)
Science	26 (50.0)	8 (28.6)	18 (75.0)
Public health programme			
Minor in public health	39 (75.0)	23 (82.1)	16 (66.7)
Second major in public health	13 (25.0)	5 (17.9)	8 (33.3)
Mean age in years (Standard deviation)	21.6 (2.7)	21.3 (1.8)	21.9 (3.4)

Table 2 details participants’ perceptions of interdisciplinary learning based on the frequency of exposure. Overall, participants had positive views on interdisciplinary learning, with over 40% expressing favourable sentiments in five out of seven statements. The strongest perception was regarding the importance of interdisciplinary learning for a future career in public health, with 67.3% of participants rating it as either *“to a high extent”* or *“to a very high extent.”* Among those frequently exposed to interdisciplinary learning, 91.7% rated its importance as *“to a high extent”* or *“to a very high extent,”* compared to 46.4% of those with infrequent exposure.

**Table 2:** Perceptions of interdisciplinary learning based on frequency of exposure.

Perception statement	Total	Infrequent exposure	Frequent exposure	*p*-value
	(*N* = 52)	(*N* = 28)	(*N* = 24)	
	*n* (%)	*n* (%)	*n* (%)	
1. Extent of shared competencies between the public health educational programmes and the student’s first major				
Not at all/To a low extent/To some extent	32 (61.5)	26 (92.9)	6 (25.0)	<0.001
To a high extent/To a very high extent	20 (38.5)	2 (7.1)	18 (75.0)	
2. Extent of the ability to draw connections between the competencies acquired from the public health educational programmes and the student’s first major, and vice versa				
Not at all/To a low extent/To some extent	27 (51.9)	25 (89.3)	2 (8.3)	<0.001
To a high extent/To a very high extent	25 (48.1)	3 (10.7)	22 (91.7)	
3. Extent of satisfaction with the current level of interdisciplinary learning between the public health curriculum and the student’s first major curriculum				
Not at all/To a low extent/To some extent	28 (53.9)	21 (75.0)	7 (29.2)	0.001
To a high extent/To a very high extent	24 (46.1)	7 (25.0)	17 (70.8)	
4. Extent to which lecturers in the public health educational programmes incorporated elements of interdisciplinary learning into their courses				
Not at all/To a low extent/To some extent	29 (55.8)	22 (78.6)	7 (29.2)	<0.001
To a high extent/To a very high extent	23 (44.2)	6 (21.4)	17 (70.8)	
5. Extent to which lecturers in the student’s first major programme incorporated elements of interdisciplinary learning into their courses				
Not at all/To a low extent/To some extent	37 (71.2)	23 (82.1)	14 (58.3)	0.07
To a high extent/To a very high extent	15 (28.8)	5 (17.9)	10 (41.7)	
6. Extent to which interdisciplinary learning is considered important for a future career in public health				
Not at all/To a low extent/To some extent	17 (32.7)	15 (53.6)	2 (8.3)	0.001
To a high extent/To a very high extent	35 (67.3)	13 (46.4)	22 (91.7)	
7. Extent to which interdisciplinary learning is considered important for a future career in the student’s first major				
Not at all/To a low extent/To some extent	22 (42.3)	16 (57.1)	6 (25.0)	0.02
To a high extent/To a very high extent	30 (57.7)	12 (42.9)	18 (75.0)	

Participants’ frequency of exposure to interdisciplinary learning was significantly associated with several factors: the extent of shared competencies between the public health educational programmes and their first major, the ability to draw connections between public health and their first major (and vice versa), satisfaction with the current level of interdisciplinary learning, the extent to which lecturers in the public health educational programmes incorporated interdisciplinary learning into their courses, and the perceived importance of interdisciplinary learning for future careers in both public health and their first major (all *p*-values <0.05). However, participants’ frequency of exposure to interdisciplinary learning was not significantly associated with the extent to which lecturers in their first major programme incorporated interdisciplinary learning into their courses (*p* = 0.07).

### Qualitative results

**Figure 1:**
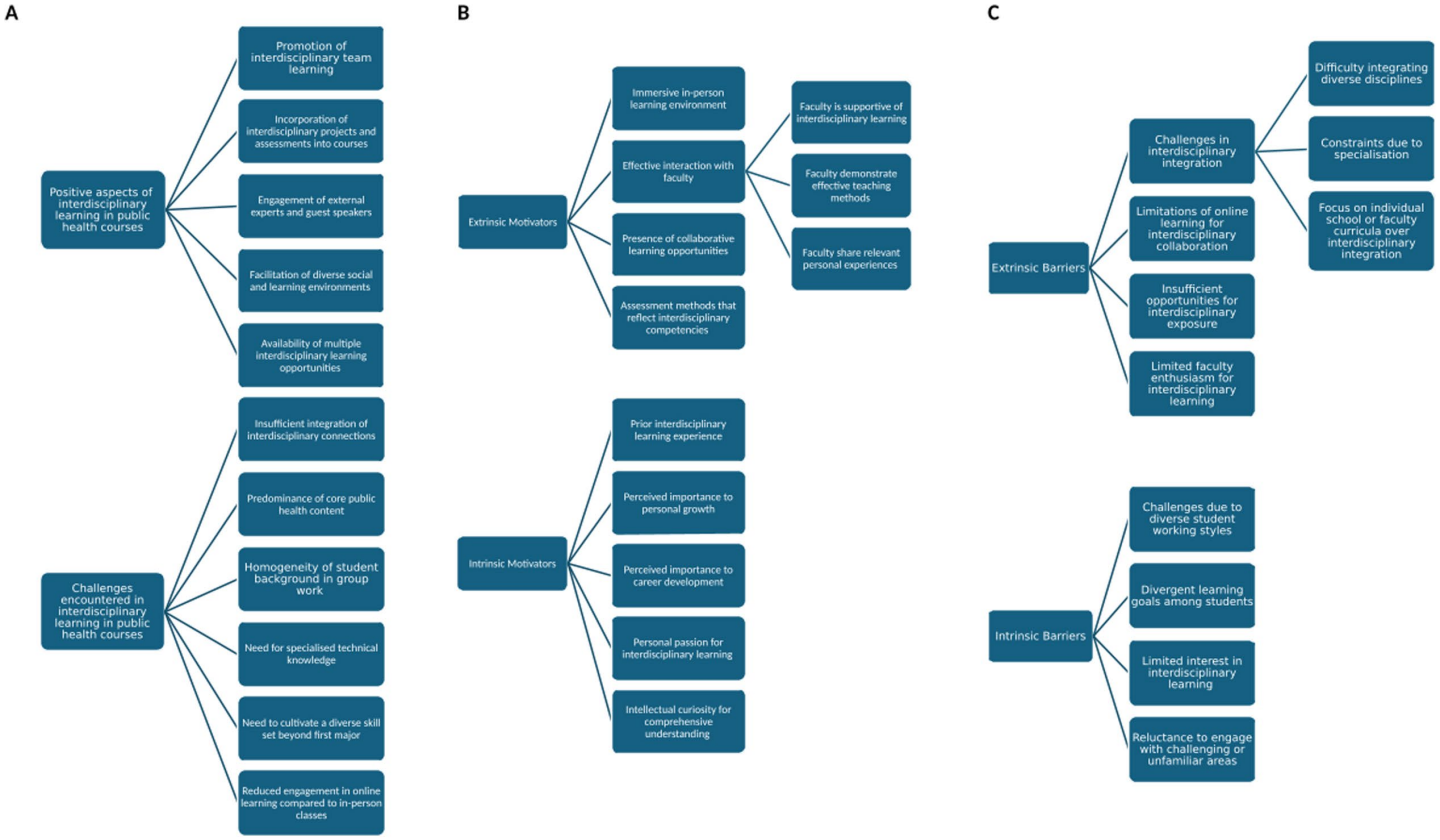
**(A)** Themes and subthemes related to interdisciplinary learning experiences under the public health educational programmes. **(B)** Themes and subthemes on the facilitators to interdisciplinary learning. **(C)** Themes and subthemes on the barriers to interdisciplinary learning.

A total of 11 students participated in the IDIs. Table 3 presents the characteristics of these participants. Figure 1 illustrates (A) the themes and subthemes related to interdisciplinary learning experiences within the public health educational programmes, (B) the themes and subthemes concerning facilitators of interdisciplinary learning; and (C) the themes and subthemes addressing barriers to interdisciplinary learning. Supplementary Tables 1a–c provide the corresponding illustrative quotes.

**Table 3:** Demographic characteristics of participants in in-depth interviews.

Demographic characteristic	Total
	(*N* = 11)
	*n* (%)
Sex	
Male	1 (9.1)
Female	10 (90.9)
Age (years)	
18–20	2 (18.2)
21–24	9 (81.8)
Year of study	
Junior (Year 1 and 2)	1 (9.1)
Senior (Year 3 and above)	10 (90.9)
Faculty/school	
Non-science	7 (63.6)
Science	4 (36.4)
Public health programme	
Minor in public health	9 (81.8)
Second major in public health	2 (18.2)

### Experiences with interdisciplinary learning in the public health educational programmes

A majority of the students reported that interdisciplinary projects and assessments were incorporated into public health courses. For instance, one student noted, *“In one Global Health module, we had to create a communication plan with infographics. This assessment required marketing skills, which I could leverage from my Business School experience, making it easier for me to apply what I had learned”* (S3). Other students also appreciated the variety of interdisciplinary learning opportunities available. For example, one student mentioned, *“There were many opportunities, such as an internship at a public hospital where I worked on both public health and other disciplinary tasks. Additionally, a public health conference provided excellent networking opportunities where I interacted with people from diverse backgrounds and disciplines”* (S9).

Students valued the positive experiences from an environment that promotes interdisciplinary learning, such as inviting external speakers from diverse fields to teach or guest lecture in public health courses. As one student commented, *“In the lifestyle behaviour module, having people from the Health Promotion Board was valuable. They could discuss epidemiology and related topics from their field, which enhanced the interdisciplinary learning experience”* (S9). Furthermore, the promotion of interdisciplinary team learning enriched the overall educational experience. Another student observed, “*In Public Health, there’s a mix of majors in group work. It’s interesting because, as someone not from a Life Science background, I bring a societal perspective while others provide scientific insights. This diversity was a valuable preview of a workplace setting where such differences are needed and appreciated”* (S1).

Despite these efforts, certain challenges remain. Some students feel that the connections between public health courses and their first major may not always be clear, requiring them to independently integrate the public health knowledge with their primary field. One student expressed, *“I feel the links between public health courses and students’ first majors may not always be clearly defined. It often relies on students to make these connections and apply the knowledge”* (S1).

Additionally, the skills required for public health courses can differ significantly from those needed in students’ first majors. This disparity may necessitate the development of a broader skill set. As one student mentioned, *“The skills from Public Health, like conducting scientific research and writing research papers, are quite different from those in Business. To succeed in interdisciplinary courses, you need a diverse skill set”* (S3). Moreover, some students noted that certain public health courses did not naturally lend themselves to interdisciplinary learning, especially when specialised technical knowledge was required.

The mode of delivery for public health courses—whether online or in-person— affected the experience of interdisciplinary learning. In-person classes facilitated more spontaneous interactions and collaborations through face-to-face discussions and informal conversations, contributing to richer interpersonal and interdisciplinary experiences compared to online formats. One student shared, *“Online classes have breakout rooms, but it feels awkward because we never meet face-to-face. This impedes the interdisciplinary learning experience. I still prefer the richer experience of in-person classes”* (S4).

### Facilitators to interdisciplinary learning

Various factors that facilitate interdisciplinary learning among students have been identified, including both extrinsic and intrinsic motivators. Extrinsic motivators, such as effective interaction with faculty, were emphasised by students as key to enhancing their engagement in interdisciplinary learning, particularly when teachers were supportive. For instance, one student noted, *“I remember a public health course on biostatistics. The professor encouraged us to share from our first major’s point of view. It was interesting to see how much you can learn from the rest of the students without any statistics background, it showcases various perspectives of analysing a question”* (S10). Faculty members who demonstrated effective teaching methods also played a crucial role. As one student mentioned, *“My favourite part about taking Public Health modules is that the professors are clearly very invested in teaching. The classes are smaller, and every professor is very passionate and uses interesting teaching materials to teach the students”* (S2). Additionally, students appreciated when faculty shared relevant personal experiences.

Other than effective interaction with faculty, extrinsic motivators at the programme and university level included the presence of collaborative learning opportunities and assessment methods that reflect interdisciplinary competencies, which further supported their learning. One student highlighted, *“The main interdisciplinary learning I had was through group work that involved a video project. This was interesting because it was my first module in Life Sciences at the university, and the assessment tested our knowledge and skills beyond my first major”* (S11).

Intrinsic motivators were critically important as well, especially the perception of interdisciplinary learning as valuable for career development. One student emphasised this, saying, *“As a future doctor, we should not only treat patients but also understand what causes diseases. For example, in an interdisciplinary project with Public Health, we learn what causes diseases in the first place, unlike in Biology where we only learn about the disease itself”* (S8). Prior experience with interdisciplinary learning was another important facilitator. Intellectual curiosity for comprehensive understanding also motivated students. One student shared, *“I’ve come to appreciate interdisciplinary learning because some issues cannot be solved through just one discipline, like Social Science or Biology. For example, I’m interested in zoonotic diseases, and my essay on bird flu showed that you cannot just look at it from a Public Health perspective. You need to understand the social and local community implications. Interdisciplinary learning provides different insights into complex issues, making it essential for understanding health and its intersections with technology, medicine, food, and more”* (S1). Additional intrinsic facilitators included the perceived importance of interdisciplinary learning for personal growth and personal passion for the subject.

### Barriers to interdisciplinary learning

Various factors that impede interdisciplinary learning among students have also been identified, including both extrinsic and intrinsic barriers. A prominent extrinsic barrier at the programme and university level was the difficulty in integrating diverse disciplines. One student highlighted this issue, stating, *“A typical class often has people from different majors, it’s very hard to integrate all these majors into Public Health while also focusing on Public Health”* (S9). Constraints due to specialisation were also noted, as explained by another student, *“As I’ve moved from level 1 K to 3 K modules, the focus shifts from more interdisciplinary content to less because I’m advancing in depth of knowledge. In introductory modules, you learn about topics like climate change from multiple perspectives, such as genetically modifying mosquitoes to prevent dengue. However, at higher levels, the focus becomes more in-depth on specific areas, such as Neurosciences, which reduces interdisciplinary overlap”* (S11). Additionally, the emphasis on individual school or faculty curricula over interdisciplinary integration was a significant barrier. One student commented, *“Firstly, there is a lack of understanding of what other majors are doing. For example, Public Health professors might need to ask me what I do in my business curriculum. Within the school or faculty, there’s not much effort to integrate different curriculums. Each school has its own dean and management office focusing on their own curriculums, rather than trying to intentionally link with others”* (S2).

Intrinsic barriers were also persistent for some students. Challenges arose from diverse working styles within groups. One student described, *“It was highly affected by mixing different students in the same project. Difficult experiences with certain group mates, who not only knew little about interdisciplinary learning but also had very different working styles, contributed to poor-quality work, which affected my overall experience”* (S11). Divergent learning goals among students further impacted the experience. As one student explained, “*Working with exchange students or students with different learning goals for what they want from the module might impact the whole experience. Exchange students might be here more for exposure and not want to spend as much time studying because they want to explore Singapore. But as a full-time university student, I want to spend more time on learning from multiple disciplines”* (S1). Other intrinsic barriers included limited interest in interdisciplinary learning and reluctance to engage with challenging or unfamiliar areas. As another student mentioned, *“Taking modules can be quite strategic as it affects grades. So, taking a module that I feel I can handle well might be more beneficial for my grades, realistically (laughs)”* (S3).

## Discussion

This mixed-methods study’s exploration of interdisciplinary learning within undergraduate public health programmes presents valuable insights that can significantly inform higher education pertaining to health professions. As public health education prepares future professionals to address complex, multifaceted challenges, fostering interdisciplinary learning is crucial for equipping students with the diverse skills essential in modern healthcare settings. Our quantitative results indicate that nearly half of the students surveyed frequently engage in interdisciplinary learning. Positive perceptions were notably higher among those with frequent exposure, particularly regarding the importance of interdisciplinary learning for future careers in public health. This finding was corroborated by qualitative data, which highlighted career development as a prominent facilitator of interdisciplinary learning. Effective interactions with faculty, collaborative learning opportunities, and diverse interdisciplinary experiences enhanced student engagement, although challenges such as integrating different disciplines and varying skill sets persisted.

The alignment between quantitative and qualitative findings underscores the value of interdisciplinary learning in higher education pertaining to health professions. The positive perception of interdisciplinary learning for future careers, observed in the quantitative survey, was corroborated by qualitative insights, which emphasised career development as a key facilitator. This consistency aligns with existing research highlighting the importance of interdisciplinary education in preparing students for complex, real-world problems requiring multiple perspectives (19). The connection between frequent exposure and factors such as shared competencies and satisfaction with interdisciplinary efforts further highlights the significance students place on these learning experiences. As healthcare increasingly demands collaborative, cross-disciplinary approaches, students’ experiences of interdisciplinary learning are directly linked to their ability to navigate diverse professional environments. This aligns with broader discussions in health education, where interdisciplinary competence is recognised as essential for tackling complex public health issues and delivering patient-centred care (19). The significance of interdisciplinary exposure in shaping students’ satisfaction and shared competencies further reinforces the necessity of such approaches in preparing health professionals.

Students’ experiences of interdisciplinary learning in undergraduate public health programmes reveal both notable successes and critical challenges. Positive interdisciplinary experiences, such as projects involving communication plans that utilise skills from other fields like marketing, provided students with opportunities to develop communication strategies critical to health promotion and education. This reflects a broader trend in health education, which incorporates skills from diverse disciplines, such as the social sciences and communication, to enhance problem-solving and leadership abilities (20). The alignment of interdisciplinary approaches with real-world applications, including internships and conferences, echoes similar practices in medical and health sciences education, where experiential learning and exposure to varied perspectives are critical for student development (21). However, the benefits of these experiences are not uniform. Despite integrating external speakers and diverse perspectives into public health courses, students reported a lack of clear connections between the public health courses and their primary majors. This disconnect suggests a need for more deliberate and structured efforts to bridge these disciplines, indicating a gap in curricular design that could hinder the full potential of interdisciplinary learning.

The disparity between the skills required for public health and those from students’ primary majors complicates the interdisciplinary learning experience. Students noted that specialised skills in public health, such as scientific research and data analysis, often contrasted sharply with those used in their first majors. This is a common issue in higher education pertaining to health professions, where students often struggle to reconcile the distinct demands of specialised health fields, such as epidemiology or clinical practice, with broader interdisciplinary competencies (5). Addressing this challenge calls for curricular designs that not only introduce interdisciplinary content but also provide structured support to help students navigate these transitions, ensuring health profession students can apply diverse skills in cohesive and meaningful ways across disciplines.

The mode of delivery is also crucial in the experience of interdisciplinary learning in higher education pertaining to health professions. Students expressed a preference for in-person learning, emphasising the value of face-to-face interactions for fostering collaboration and spontaneous discussions—critical elements for effective interdisciplinary education in healthcare. The challenges of online learning environments, such as awkward interactions in breakout rooms, highlight the need for improved online teaching strategies that can replicate the benefits of face-to-face engagement. Research supports the benefits of in-person discussions for facilitating interdisciplinary learning, emphasising the importance of face-to-face interactions in fostering a collaborative and engaging social learning environment (22). Adopting blended learning approaches that combine in-person and online elements, based on Universal Design for Learning (UDL) principles, could enhance interdisciplinary engagement in higher education pertaining to health professions (23). This strategy would create inclusive learning environments where all students, regardless of learning preferences or physical location, can effectively engage with interdisciplinary content, preparing them for diverse roles in healthcare.

The exploration of facilitators of interdisciplinary learning reveals a complex interplay of extrinsic and intrinsic factors. Among the extrinsic facilitators, supportive faculty who encourage students to share perspectives from their primary disciplines are crucial. This aligns with McFarlane’s (24) findings that faculty support is essential for guiding students through interdisciplinary projects and navigating the complexities of integrating knowledge from different disciplines. Faculty who actively promote interdisciplinary discussions and projects help students develop the collaborative and problem-solving skills necessary for modern healthcare environments. These findings are consistent with research in medical and public health education, where interdisciplinary group work is seen as vital for preparing students to address multi-faceted health challenges (25). The importance of soft skills, such as communication and teamwork, further emphasises the role of interdisciplinary learning in higher education pertaining to health professions, where these skills are essential for effective healthcare delivery (20).

Intrinsic motivators driving interdisciplinary learning are equally relevant to higher education pertaining to health professions. Students’ enthusiasm for integrating multiple perspectives, particularly in understanding complex health issues, underscores the need for curricular structures that harness these motivations. When curricula align with students’ intrinsic interests, they are more likely to engage deeply with interdisciplinary content, enhancing their readiness for professional healthcare roles (26). However, when curricular design fails to reflect these intrinsic drivers, the potential for interdisciplinary learning diminishes, limiting students’ capacity to engage with the full spectrum of public health challenges they will face in their careers.

Integrating interdisciplinary approaches in public health education at the programme and university level presents notable challenges. A key issue involves aligning diverse disciplinary perspectives within a unified public health context. Students noted that the shift from broad interdisciplinary content in introductory courses to more focused specialisation in advanced courses can limit opportunities for interdisciplinary engagement. This progression may affect how effectively students integrate and apply knowledge from various fields in their public health practice. To address these challenges, it is essential to prioritise course and learning objectives that explicitly encourage students to make connections between different disciplines and reflect on the role of interdisciplinarity in their coursework and group projects. Specific strategies for integrating interdisciplinary learning include the introduction of interdisciplinary case studies, which allow students to apply knowledge from multiple fields to real-world public health challenges. Additionally, co-teaching by faculty from diverse disciplines can encourage students to experience and integrate different disciplinary approaches into their understanding of public health. Collaborative projects across various fields also provide opportunities for students to work together on public health issues, fostering a holistic approach to problem-solving. Intentional curriculum planning is crucial to map interdisciplinary elements from the start of the programme, ensuring that students engage with diverse perspectives and develop the skills needed to integrate them throughout their academic and professional lives. These strategies help enhance students’ ability to see the relevance of multiple perspectives, ultimately preparing them to address complex public health challenges more effectively (27).

While the shift from generalist to specialist content can reduce interdisciplinary overlap, it also underscores the need for a balanced educational approach. Teachers in higher education pertaining to health professions must ensure that interdisciplinary learning remains a core component even as students delve deeper into specialisations. Maintaining interdisciplinary connections is particularly critical in public health and healthcare, where professionals must integrate knowledge from various fields to address complex, interconnected health issues. Embedding interdisciplinary activities throughout the curriculum ensures that students can apply their specialised knowledge in broader healthcare contexts, preparing them to be effective, future-ready healthcare professionals (27).

Teachers also play a role in incorporating interdisciplinary elements into their classrooms, though time and content restrictions may hinder execution. The lack of integration across curricula from different majors highlights a significant barrier to interdisciplinary learning. Effective interdisciplinary education requires intentional curriculum design that bridges disciplines, a sentiment echoed by Repko et al. (28). Institutional challenges to interdisciplinary learning can be addressed by creating environments that enhance interdisciplinary coordination and communication, such as new interdisciplinary centres and targeted research funding (23).

Intrinsic barriers, including a lack of personal interest among students and diverse working styles within groups, also impede interdisciplinary learning. Differences in work habits and expectations often lead to conflicts and inefficiencies, resulting in suboptimal group performance and negatively impacting the learning experience. Research by Choi and Pak (29) underscores that cultural differences and varied academic backgrounds can hinder effective interdisciplinary collaboration. Conversely, Reich and Reich (30) argue that while intersectional perspectives introduce challenges, they also enrich interdisciplinary work. They suggest that finding common ground amid diverse group dynamics is essential, making interdisciplinary relationships more vibrant even when initial common ground seems elusive.

Divergent learning goals among students can also disrupt the usefulness of interdisciplinary projects. The contrast between students seeking practical exposure and those aiming for in-depth academic learning creates tensions that can undermine the collaborative process. Such differences highlight the need for clearer objectives and expectations for interdisciplinary projects to ensure alignment among participants and to enhance the cohesiveness of group work. Limited intrinsic interest and reluctance to engage with challenging or unfamiliar areas also contribute to barriers. As students devise strategies for selecting courses to optimise their grades, their reluctance to undertake difficult or unfamiliar subjects may result in missed opportunities for genuine interdisciplinary exploration. This reflects a broader issue where students’ academic strategies can conflict with the goals of interdisciplinary learning, suggesting the need for curricular incentives and support systems to encourage engagement with challenging interdisciplinary content. Comprehensive support systems are necessary to help students navigate these new and challenging areas.

### Recommendations and future directions

Interdisciplinary learning is essential in preparing the next generation of public health professionals to address complex challenges in the 21st century. The lack of clear connections between public health undergraduate programmes and other first major programmes creates a learning gap. Students need clearer guidance and examples from faculty, such as highlighting research areas, providing real-world applications, and using case studies to demonstrate interdisciplinary intersections. The University of Hong Kong’s case study (31) shows the importance of bridging secondary education with university expectations and having a clear curriculum vision. University administration should ensure coherence and integration across disciplines to prevent fragmentation and support career-ready skills.

Students also show strong interest in interactive sessions with external speakers and professionals, which enhance engagement and provide practical insights (32). Such methods break the monotony of traditional lectures and enrich learning. Additionally, integrating perspectives from the humanities, arts, and sciences can improve interdisciplinary outcomes, such as teamwork and communication skills (33).

Based on the motivators and barriers identified in this study, several recommendations for public health teachers and policymakers can help enhance interdisciplinary learning. Teachers should redesign curricula to include interdisciplinary components, such as co-teaching and joint projects, to encourage students to make connections across disciplines. Faculty development programmes should equip teachers with the skills to teach interdisciplinary courses effectively, including collaborative teaching techniques. Policymakers should create institutional incentives, such as funding for interdisciplinary projects and support for faculty collaboration, to promote interdisciplinary teaching and research.

Additionally, mentorship programmes and structured team-building activities can foster collaboration and improve communication among students from different disciplines. Partnerships with public health agencies can expose students to real-world interdisciplinary challenges, providing practical experience. By implementing these recommendations, public health teachers and policymakers can improve interdisciplinary learning, ensuring that students are better prepared to tackle complex, interconnected issues in their future careers.

### Strengths and limitations

There were several strengths and limitations in our current study. To the best of our knowledge, this is the first study on interdisciplinary learning in public health undergraduate programmes using a mixed methods approach, which enabled triangulation. Data saturation was also achieved in the qualitative analysis, strengthening the results of this study. However, certain limitations were present. First, during the qualitative phase, we did not show the transcripts to participants to confirm whether their responses had been accurately documented. The interviewers addressed this by regularly paraphrasing and “checking back” with participants to verify the accuracy of their responses. Second, we could not eliminate social desirability bias, as the data were self-reported. However, we have outlined in the Methods section the steps taken to minimise this bias. Third, causal relationships cannot be inferred from the cross-sectional design of the quantitative component.

## Conclusion

In conclusion, this study underscores the significance of interdisciplinary learning within undergraduate public health programmes, offering valuable insights into its impact on students’ educational and career trajectories. The positive influence of frequent interdisciplinary exposure on students’ career aspirations mirrors the evolving demands of higher education pertaining to health professions, where collaboration across disciplines is essential for addressing complex healthcare challenges. However, barriers such as disciplinary disconnects and varied skill sets highlight the need for curricular innovations that better integrate interdisciplinary elements. By addressing these gaps and enhancing faculty support, higher education pertaining to health professions can cultivate more adaptable and skilled graduates, capable of navigating the multifaceted nature of healthcare and public health practice. Aligning educational practices with students’ intrinsic motivations and ensuring a cohesive, interdisciplinary curriculum will better equip future health professionals to tackle the intricacies of their roles in an increasingly interconnected world.

## Data Availability

The original contributions presented in the study are included in the article/Supplementary material, further inquiries can be directed to the corresponding author.
