# Supplementary material for: Student perspectives on interdisciplinary learning in public health education: insights from a mixed-methods study
Source: Front Public Health. 2024 Dec 10;12:1516525. doi: 10.3389/fpubh.2024.1516525 (PMC11667116; doi:10.3389/fpubh.2024.1516525)
Supplement: Supplementary file 1 [file Table_1.docx]

**Supplementary table 1a: Illustrative quotes for the themes and subthemes related to interdisciplinary learning experiences under the public health educational programmes**

| **Theme** | **Subtheme** | **Illustrative quotes** |
| --- | --- | --- |
| Positive aspects of interdisciplinary learning in public health courses | Promotion of interdisciplinary team learning | S1: "I think especially in Public Health, there’s a lot of mix of majors. From what I’ve seen during group work, it’s very different. There are always students from other first majors. But, because I’m not a Life Science student, I realised we think very differently. They provide more of the scientific understanding, while I look at it from a societal perspective, considering different stakeholders. It was a nice preview of a diverse workplace setting where such diversity is needed and appreciated."  S10: "Most of the time, I’m there to learn from my group members. We discover more things together as a group, realising underlying issues more often. For example, we just started our group project. One of my teammates is a Sociology major, and another is a Food Science major with a second major in Humanities. They have different skills that I don’t have. We were told to perform interviews in the course, and they were efficient at scripting and phrasing questions to make the interview flow like a conversation. They also knew how to promote certain behaviours, which is something I struggle with as a Life Science major."  S5: "One Health links the health of animals, the environment, and humans. Therefore, I was able to learn from various disciplines. For example, there were lectures on Microbiology and foodborne pathogens, overlapping with what I study in Food Science & Technology." |
|  | Incorporation of interdisciplinary projects and assessments into courses | **S3:** "One Global Health module required us to create a communication plan with infographics, this assessment requires marketing skill which I can leverage from what I have learnt from Business School, so it was easy for me to apply it there."  **S9:** "In the lifestyle behaviour course, we talk about defining and perceiving health, covering biomedical and holistic views. The biomedical view is “if you are disease-free, you are healthy,” while the holistic view includes mental well-being. Integrating these views helps improve overall health. We are expected to work on group projects which should lead to holistic health improvements even though I am from Biomedical Engineering."  **S11:** "I saw interdisciplinary learning in public health looking at factors affecting human health from various perspectives like religion and gender. The assignments that we are given also reflect this.” |
|  | Engagement of external experts and guest speakers | S9: "For the lifestyle behaviour module, bringing in people from the Health Promotion Board is valuable because when they are from that field, they can talk about epidemiology and topics related to other disciplines. So, I would say it's a positive interdisciplinary learning experience."Top of FormBottom of Form |
|  | Facilitation of diverse social and learning environments | **S10:** "The professor told us that all of us were from different majors, either minoring or second majoring in Public Health. We all had different first disciplines, so we shared our perspectives from Life Sciences, Environmental Science, or Food Science majors on a case study. We all had different perspectives towards the problem. The professor was able to create a conducive environment to facilitate such interdisciplinary discussions." |
|  | Availability of multiple interdisciplinary learning opportunities | **S9:** "There were many opportunities available. I applied for an internship at a public hospital, where I worked on public health and other disciplinary tasks. There was also a public health conference, which was a great networking event. I volunteered to help and met and interacted with many people from diverse backgrounds and disciplines." |
| Challenges encountered in interdisciplinary learning in public health courses | Insufficient integration of interdisciplinary connections | **S1:** "I feel the connections between public health courses and students' first major may not always be clearly defined. Often, it is up to the students to take the initiative to make these links and apply the knowledge." |
|  | Predominance of core public health content | **S3:** "I think… at the end of the day, what I learn in Public Health is very different from Business. For public heath courses, there’s still quite a bit of content that is solely related to Public Health." |
|  | Homogeneity of student background in group work | **S4:** "I feel that there isn’t much diversity in the group work, considering that most of the Public Health students are from Life Science."  **S11:** "The fact that there are a lot of group projects makes it a good opportunity to interact with people, but generally, people who want to do Public Health often come from the Life Sciences. Probably for reasons similar to myself, or having a general interest in Public Health." |
|  | Need for specialised technical knowledge | **S3:** "I think the course on epidemiology really pushed me out of my comfort zone because it was quite technical rather than interdisciplinary compared to what I’m used to in Business School. Over there, I had to rely a lot more on my group members to clarify things or ask questions. Many of them were from Life Science and had a lot of knowledge in epidemiology, so they could quickly come up with solutions for the project, while I needed more guidance in the process." |
|  | Need to cultivate a diverse skill set beyond first major | **S3:** "Because the skills that I get from Public Health are very different from those in Business, like learning how to do scientific research and structure research papers, etc. Therefore, I feel to take interdisciplinary courses in public health, you need to have a broad skill set." |
|  | Reduced engagement in online learning compared to in-person classes | **S4:** "Yeah, online classes have breakout rooms, but it feels awkward because we never see the person face-to-face, which impedes the interdisciplinary learning experience. I still prefer the interdisciplinary learning experience I had in in-person classes." |

**Supplementary table 1b: Illustrative quotes for the themes and subthemes on the facilitators to interdisciplinary learning**

| **Theme** | **Subtheme** | **Sub subtheme** | **Illustrative quotes** |
| --- | --- | --- | --- |
| Extrinsic motivators | Immersive in-person learning environment |  | **S1:** "First of all, having in-person classes facilitates interdisciplinary learning as opposed to online learning. I think there are benefits to doing everything in person."  **S9:** "We also had this thing called live coding. During the class, you do coding in front of the teacher, and if you have any questions, you can ask on the spot. It’s about that week's topic. Doing this every week really helps." |
|  | Effective interaction with faculty | Faculty is supportive of interdisciplinary learning | **S1:** "I mentioned the professors are supportive when you decide to pursue a more interdisciplinary route. Their specialisation is more grounded in the discipline, like on the environmental side, they are more knowledgeable about how rocks or soil work. Even if I want to pursue interdisciplinary areas that they’re not familiar with, they are supportive. So, there’s a need for more expert guidance, but they are definitely supportive of pursuing interdisciplinary learning."  S10: "I remember a public health course on biostatistics. The professor encouraged us to share from our first major’s point of view. It was interesting to see how much you can learn from the rest of the students without any statistics background, it showcases various perspectives of analysing a question." |
|  |  | Faculty demonstrate effective teaching methods | S10: "The way the slides were presented by the professor in the Public Health content was very similar to my initial learning experience in Epidemiology. The content was not very in-depth but was intuitive and well-defined, making it easy to understand even without prior knowledge."  S2: "My favourite part about taking Public Health modules is that the professors are clearly very invested in teaching. The classes are smaller, and every professor is very passionate and uses interesting teaching materials to teach the students." |
|  |  | Faculty share relevant personal experiences | S8: "Many examples are actually anecdotes. One professor talked about her mother, who had a mental illness and how she was helped with Valium. This made it memorable and shows that many professors use their anecdotes in teaching." |
|  | Presence of collaborative learning opportunities |  | S10: "The professor told us that we were all from different majors since we were Minoring or Second Majoring in Public Health. We had diverse first disciplines, and we were encouraged to share our perspectives." There were Life Sciences majors, Environmental Science majors, and a Food Science major. We all had different perspectives towards the problem."  S11: "Working with people from different disciplines like Psychology, Economics, Physics, and Mathematics has been valuable. For example, in one of my modules, we discussed topics where my Life Sciences perspective focused on animals, trees, and plants, while my Physics colleague focused on the underlying processes. This exchange of perspectives is an enriching part of interdisciplinary learning for me." |
|  | Assessment methods that reflect interdisciplinary competencies |  | S11: "The main interdisciplinary learning I had was through group work that involved a video project. This was interesting because it was my first module in Life Sciences at the university, and the assessment tested our knowledge and skills beyond my first major." |
| Intrinsic motivators | Prior interdisciplinary learning experience |  | S9: "I have done other similar interdisciplinary modules before like BN3101. It's a design module where we speak to clinicians who set up their problems and needs. We go through stages like problem identification, needs specification, intellectual property, and regulatory affairs. After getting their input, we do deliverables for each stage. For example, in physiotherapy-related projects, speaking to the university hospital physiotherapists gives us more insights. So, I am not new to interdisciplinary learning."  S11: "I am part of the College of Humanities and Science, interdisciplinary learning has been part of my experience since the beginning of university and thus I am familiar with it." |
|  | Perceived importance to personal growth |  | S1: "I guess first and foremost there's a lot of soft skill building because I think soft skills and communication are very necessary to work in fields that are trying to solve problems, which I guess is almost every field. Learning to negotiate differences is something very common in interdisciplinary teams or groups. I think I'm still trying to figure it out, but that experience has helped me grow as a person and become a better version of myself." |
|  | Perceived importance to career development |  | S8: "As a future doctor, we should not only treat patients but also understand what causes diseases. For example, in an interdisciplinary project with Public Health, we learn what causes diseases in the first place, unlike in Biology where we only learn about the disease itself."  S10: "One example is disease control for dengue. As a Life Scientist, I can focus on the scientific aspect, like the pathogen, but to solve the issue, I need interdisciplinary ideas. For instance, understanding behaviours from a humanities perspective can help control breeding in an area. Another example is hawker management to control foodborne diseases, where we need to consider economic and practical perspectives to find viable solutions."  S9: "Interdisciplinary learning is very crucial, especially in university education. Even in the first major itself, like in Biomedical Engineering, the first two years involve building foundational topics. You may need to take modules like Organic Chemistry and machine learning, and at that time, you might wonder why you're doing it. But later, you realise its usefulness in projects where Chemistry or coding is required. Interdisciplinary learning is important because it focuses on holistic learning, preparing you for your future career." |
|  | Personal passion for interdisciplinary learning |  | S1: "I took Public Health because I realised during the onset of COVID that healthcare is a big and important field. I wanted to understand more about health from a different perspective as a social scientist. We study in a social manner, but I decided to take Public Health to complement that with a more scientific or epidemiological approach to health issues and pair it with what I learned from my first major." |
|  | Intellectual curiosity for comprehensive understanding |  | S1: "I’ve come to appreciate interdisciplinary learning because some issues can't be solved through just one discipline, like Social Science or Biology. For example, I’m interested in zoonotic diseases, and my essay on bird flu showed that you can't just look at it from a Public Health perspective. You need to understand the social and local community implications. Interdisciplinary learning provides different insights into complex issues, making it essential for understanding health and its intersections with technology, medicine, food, and more." |

**Supplementary table 1c: Illustrative quotes for the themes and subthemes on the barriers to interdisciplinary learning**

| **Theme** | **Subtheme** | **Sub subtheme** | **Illustrative quotes** |
| --- | --- | --- | --- |
| Extrinsic barriers | Challenges in interdisciplinary integration | Difficulty integrating diverse disciplines | S9: "A typical class often has people from different majors, it's very hard to integrate all these majors into Public Health while also focusing on Public Health."  S10: "It was a module where students discussed different Public Health policies in different countries based on the severity of issues in those countries. But we didn't bring in the Economics point of view or Science point of view; it was completely focused on why a certain programme is built the way it is and how it addresses that country's issue. I couldn't bring in the perspectives of other disciplines because the module was not designed to integrate them and did not require us to do so." |
|  |  | Constraints due to specialisation | S11: "As I've moved from level 1K to 3K modules, the focus shifts from more interdisciplinary content to less because I’m advancing in depth of knowledge. In introductory modules, you learn about topics like climate change from multiple perspectives, such as genetically modifying mosquitoes to prevent dengue. However, at higher levels, the focus becomes more in-depth on specific areas, such as Neurosciences, which reduces interdisciplinary overlap." |
|  |  | Focus on individual school or faculty curricula over interdisciplinary integration | S2: "Firstly, there is a lack of understanding of what other majors are doing. For example, Public Health professors might need to ask me what I do in my business curriculum. Within the school or faculty, there’s not much effort to integrate different curriculums. Each school has its own dean and management office focusing on their own curriculums, rather than trying to intentionally link with others." |
|  | Limitations of online learning for interdisciplinary collaboration |  | S1: "My first major is Geography, which is quite interdisciplinary because it combines physical and social elements to understand issues. Many of my modules involve group work, going into target areas for interviews and surveys. In Public Health modules, if they had been conducted in person, I believe there would have been more interdisciplinary learning. However, because of COVID, they were kept online, which hindered that." |
|  | Insufficient opportunities for interdisciplinary exposure |  | S9: "I would only speak to the same person and supervisor, and we were both working on breast cancer-related projects. I didn't learn much about the other machinery. The only time I learned something new from another discipline was when a colleague came in, and we made friends. He would tell us about his project, which involved shining light into leaves to see the content of a certain chemical in agriculture. These were things I wouldn't learn from another discipline unless there were opportunities." |
|  | Limited faculty enthusiasm for interdisciplinary learning |  | S1: "Not all professors are keen on interdisciplinary learning. Some may choose to focus only on their specialisation. That’s their expertise. If I want to pursue interdisciplinary areas, they might not be interested or even familiar with them. Thus, there is a need for more expert guidance." |
| Intrinsic Barriers | Challenges due to diverse student working styles |  | S8: "Like my friends from Maths doing the project in the course. It's very new and completely different for some of them, and they haven’t worked in teams in a long time. So, teaming up now, it was such chaos, such a mess, that I had to step up as a leader."  S11: "It was highly affected by mixing different students in the same project. Difficult experiences with certain group mates, who not only knew little about interdisciplinary learning but also had very different working styles, contributed to poor-quality work, which affected my overall experience." |
|  | Divergent learning goals among students |  | S1: "Working with exchange students or students with different learning goals for what they want from the module might impact the whole experience. Exchange students might be here more for exposure and not want to spend as much time studying because they want to explore Singapore. But as a full-time university student, I want to spend more time on learning from multiple disciplines." |
|  | Limited interest in interdisciplinary learning |  | S10: "I think, most students might just want to focus on their first major and not want or not keen to learn about interdisciplinary content." |
|  | Reluctance to engage with challenging or unfamiliar areas |  | S3: "Taking modules can be quite strategic as it affects grades. So, taking a module that I feel I can handle well might be more beneficial for my grades, realistically (laughs)."  S9: "I think some of the modules were manageable because I had taken Physics, Biology, and Chemistry modules during my time in junior college, so those were quite okay for me. However, I had not taken any computing modules or classes before, so I had no background in computing or machine learning. Therefore, when I took an interdisciplinary course that included computing and machine learning, I was very overwhelmed. Given this, I feel that not many students would choose modules they are uncomfortable with." |
